# The Extensive Usage of the Facial Image Threshing Machine for Facial Emotion Recognition Performance

**DOI:** 10.3390/s21062026

**Published:** 2021-03-12

**Authors:** Jung Hwan Kim, Alwin Poulose, Dong Seog Han

**Affiliations:** School of Electronic and Electrical Engineering, Kyungpook National University, 80 Daehak-ro, Buk-gu, Daegu 41566, Korea; jkim267@knu.ac.kr (J.H.K.); alwinpoulosepalatty@knu.ac.kr (A.P.)

**Keywords:** facial emotion recognition (FER), autonomous driving, convolution neural network (CNN), Xception, ResNet, MTCNN, FER 2013 Dataset, CK+ Dataset

## Abstract

Facial emotion recognition (FER) systems play a significant role in identifying driver emotions. Accurate facial emotion recognition of drivers in autonomous vehicles reduces road rage. However, training even the advanced FER model without proper datasets causes poor performance in real-time testing. FER system performance is heavily affected by the quality of datasets than the quality of the algorithms. To improve FER system performance for autonomous vehicles, we propose a facial image threshing (FIT) machine that uses advanced features of pre-trained facial recognition and training from the Xception algorithm. The FIT machine involved removing irrelevant facial images, collecting facial images, correcting misplacing face data, and merging original datasets on a massive scale, in addition to the data-augmentation technique. The final FER results of the proposed method improved the validation accuracy by 16.95% over the conventional approach with the FER 2013 dataset. The confusion matrix evaluation based on the unseen private dataset shows a 5% improvement over the original approach with the FER 2013 dataset to confirm the real-time testing.

## 1. Introduction

A machine that precisely identifies a driver’s emotional expression is one way to reduce the number of fatal vehicle accidents. Ismail et al. [1] claimed that angry driving behavior increases the risk of a car accident and could become life-threatening to others. A facial emotion recognition (FER) system might help prevent fatal accidents and save someone’s life from enraged drivers.

Presently, many researchers of FER have been rigorously improving new FER algorithms with the aim of achieving ideal FER system performance, but few have published newly collected FER datasets. Most researchers propose their customized FER algorithms be tested with datasets such as FER 2013 [2] and extended Cohn and Kanade (CK+) [3]. FER 2013 and CK+ datasets are well-known and popular datasets. Unfortunately, neither have a sufficient amount of data compared with the CIFAR-10 dataset [4] which has 60,000 object images for training and testing. Most algorithms have reached 99% validation accuracy in recent years. Many FER datasets do not have as large a number of facial images as the well-balanced number of the CIFAR-10 dataset. Krizhevsky et al. [5], who designed and built the AlexNet algorithm, justified that the data-augmentation technique could mostly solve the overfitting problem from the small number of facial images for training FER systems. Still, in an article by Sakai et al. [6], collecting many brain signals showed better performance than using a small number of biological signals by applying the data-augmentation technique. Data augmentation seems to be a temporary solution if we do not have sufficient FER datasets in the FER system. However, a small number of facial images still failed to generalize the dataset and cause poor performance in real-time testing even with data augmentation.

Generally, relying on obtaining datasets without any rigorous inspection could cause the trained model to become disoriented by some malicious data inputs. Some images were not relevant to facial images or misplacing them into the wrong labeled directories. Removing irrelevant facial images from the FER 2013 dataset must be done to reduce unnecessary computation. Considering the number of available facial images in the dataset, removing any facial images reduced the amount of available training and testing datasets and caused an imbalanced number of each directory’s facial images. We merge with different existing datasets or collect the additional datasets to have the additional facial images for training and testing to substitute the removed facial images. Merging the different existing datasets requires a unique pre-processing technique because all datasets have different sizes or different positions of facial images on a massive scale.

Furthermore, obtaining highly qualified datasets is even more challenging than improving the FER system performance by giving lists of datasets [7]. Obtaining face images was difficult, as the FER dataset often could not access individual institutions or did not share in public due to protecting intellectual property or personal privacy. Initially, we planned to collect volunteer facial expressions without any stimulating environment. We captured random facial expressions made by themselves in front of their camera. Cowen and Keltner [8] proposed how to capture 27 distinct emotions. Their volunteers made different facial expressions based on what they saw in different emotional or provoking content. We compared the facial expression from our volunteers and the real-time facial expression from people on YouTube. People’s emotional expressions on YouTube are quite different from our volunteer acting expressions, and even from other obtained facial images from FER 2013 and CK+ datasets. If we ask volunteers to express their emotions without real-time simulated environments, the trained model based on those volunteer facial expressions will be less likely to be recognized as vital facial expressions in the real-time situation. On top of that, collecting facial images for training an FER dataset is time-consuming due to the standardizing each captured facial image size and the lack of a variety of volunteers.

We propose to operate a facial image threshing (FIT) machine, and the significant contributions to this paper to address these problems are as follows:

- We implemented the proposed FIT machine, which can collect the massive number of facial images from videos.
- The proposed FIT machine can eliminate the irrelevant facial images from the FER 2013 dataset.
- The proposed FIT machine can correct the misplaced face data in a wrong categorical class.
- The proposed FIT machine can remove the background segment but leave the facial segment.

The major applications of the proposed FIT machine are face detection [9], security [10], malware threat detection [11], and body pose estimation [12] as well as collecting the face emotions. The face detection could detect any face from lost children to criminals as the FIT machine can collect faces for storage of the dataset, and later use them for model training. The FIT machine could be handy for apprehending runaway criminals. It also could be applicable for detecting authorized personnel to access certain buildings without any identification device. The FIT machine could act as a malware threat detection when unauthorized personnel breach the system.

The rest of this paper is organized as follows. Section 2 refers to the related work of collecting facial images, pre-processing techniques, and the conventional FER datasets. Section 3 proposes and demonstrates the structure and operation of the proposed FIT machine. Section 4 analyzes the experimental results. Section 5 concludes this paper with some concluding remarks.

## 2. Related Work

In this section, we describe conventional approaches to managing FER datasets. We discuss the existing FER system challenges when the system uses FER 2013 and CK+ datasets.

McDuff et al. [13] collected 168,359 video frames from 242 facial videos in 2013. They also included the active units (AU), landmarks, labels, and other assisting properties onto their collected dataset. The team developers recorded the online visitor facial expressions while watching a short video clip to trigger their facial emotional expressions. The policy rules before accessing the website indicated that the users consent to being recorded for the team developer research or not. Online visitors must consent to the policy rules before they access the website. McDuff et al. [13] published their dataset to share in public, and the datasets can be found at the online website address. Nevertheless, their proposed approach might be quite time-consuming if the web users were unwilling to share their profile or mostly not participate in the website.

The most popular dataset is FER 2013 for many FER researchers. The FER 2013 dataset could be obtained in a comma-separable value (CSV) format or actual image. Tümen et al. [14] experimented with a convolution neural network for training the FER model. They achieved 57.1% validation accuracy with the FER 2013 dataset. However, their achieved validation accuracy was not sufficient for potential performance because even a simple convolution neural network could achieve more than 59%. Moreover, they did not inspect the actual facial images in the FER 2013 dataset thoroughly because we spotted that some malicious facial contents hinder the FER system performance. We emphasize how important it is to manage the existing dataset and increase the available number of face images to train the FER model. Another FER researcher, Zahara et al. [15], who used the FER 2013 dataset, achieved 65% of validation accuracy of the FER performance with the FER 2013 dataset. They also display the confusion matrix and the real-time performance. However, their approaches of FER 2013 are not sufficient to analyze the FER 2013 dataset. They used a face detection system based on the Haar cascade classifier method. The Haar cascade classifier method was proposed by Viola et al. [16] in 2001. Historically, computing from each colored pixel into the extracted features was considerably expensive. The computation of the Haar cascade classifier method was reduced by training the AdaBoost algorithm [17]. The Haar cascade classifier method is used not only in the face detector but also in objects. However, the proposed face detection from Zahara et al. is outdated because the Haar cascade classifier method was introduced in 2001, even before introducing the AlexNet algorithm. The Haar cascade classifier is still shallow compared with our current algorithms that we used. Their proposed facial detection was not highly accurate and can detect more irrelevant pictures of the face, confusing the pre-trained FER model during the training and testing.

Another popular FER dataset is the CK+ dataset. Before training and testing the FER model, all facial segments from the images must be extracted and resized from the original images because each image of CK+ has a larger background segment than a face. Otherwise, the unnecessary background pixels cause the FER model to be poorly trained and slow down the training speed. Despite the small number of facial images from the CK+ dataset, the performance results outperformed the FER 2013 dataset by applying the data-augmentation technique. If the face images were properly extracted before the training, the trained Xception algorithm performance generally showed between 86% and 88% if the split ratio of the training and the testing dataset is 70 to 30. Kim et al. [18] claim that the data-augmentation technique resolves a small amount of data from the CK+ dataset. They achieved over 90% validating accuracy, and the ratio of training and testing was 90 to 10. Nevertheless, they do not show or demonstrate real-time performance. Their results only display the estimated validation accuracy during the training process. Similarly, Liu et al. [19] claim that their FER performance show improvement without demonstrating the absolute performance from a different set of data. The real-time performance or unseen testing dataset is required to show the absolute performance of the FER system. Although a validation accuracy is more than 90% with a small amount of face data during the FER model training, the real-time performance is still low, as we discovered. Lucey et al. [3], who created and shared the CK+ dataset, admitted that they required a massive amount of the FER data to have a robust performance of real-world emotion classification.

After these problems from recent FER researchers, we cautiously emphasize how inspecting training and testing data is heavily influential on FER performance. In our approach, the FIT machine can solve these challenges and manage the FER datasets efficiently. The FIT machine collected 8173 facial images even without bringing volunteers to participate in our laboratory. We used the current face detected system, called the multi-task cascade neural network (MTCNN) [20], which is better performance than the Haar cascade classifier method. We mention the confusion matrix evaluation from the unseen private testing dataset of FER 2013 to confirm the estimated real-time performance and prove how a small number of face images as CK+ shows an unsatisfactory real-time performance. Using the FIT machine, a small number of datasets in the CK+ dataset become a supplement for merging the different FER datasets.

## 3. Proposed FIT Machine

**Figure 1:**
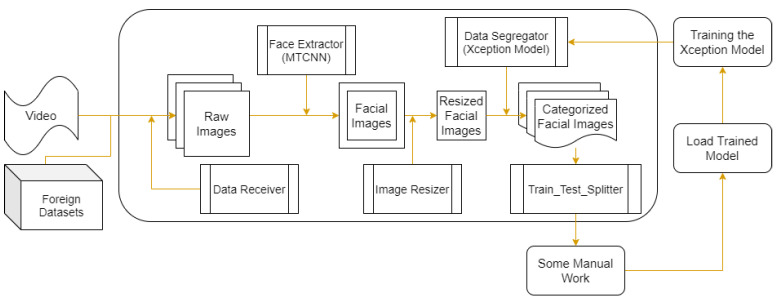
Proposed FIT machine diagram.

The FIT machine [21] from Figure 1 coverts a recorded video clip or any foreign datasets into facial images as a part of our desirable FER training and testing datasets. In other words, once a downloaded YouTube video clip or any foreign datasets enter the FIT machine, the FIT machine starts to convert from a video as an input into the cropped, resized, and categorized facial images as the output. The FIT machine consists of the data receiver, the face detector as the MTCNN, the image resizer [22], and the data segregator as the pre-trained Xception algorithm model [23].

In the FIT machine, the data receiver converted video into images. Suppose we set 20 frames per second from a 2-min video clip, the data receiver generated around 2400 images. After a recorded video went through the data receiver, the face extractor as MTCNN, which was introduced by Zhang et al. [20], detected and extracted the facial image parts of each video frame. MTCNN is obtainable via typing “pip install MTCNN” without implementing it from scratch. If the data receiver had a foreign dataset as a comma-separable value (CSV) format or raw images, it converts them into images before entering MTCNN as the face extractor.

**Figure 2:**
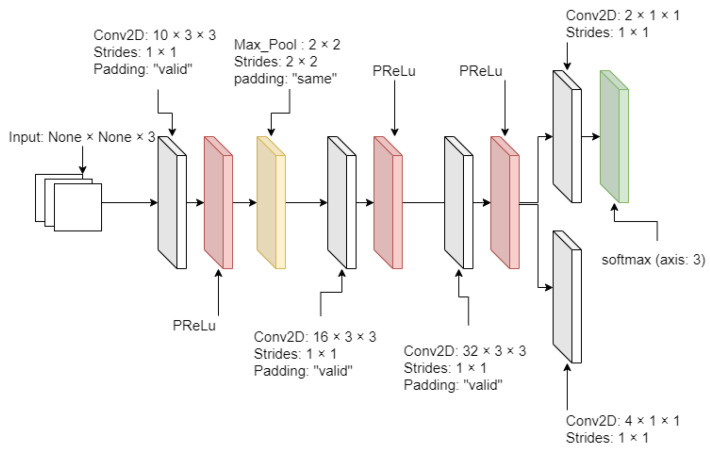
P-Net diagram.

**Figure 3:**
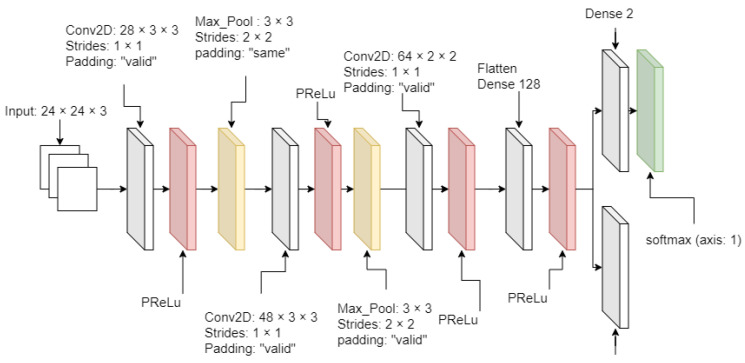
R-Net diagram.

**Figure 4:**
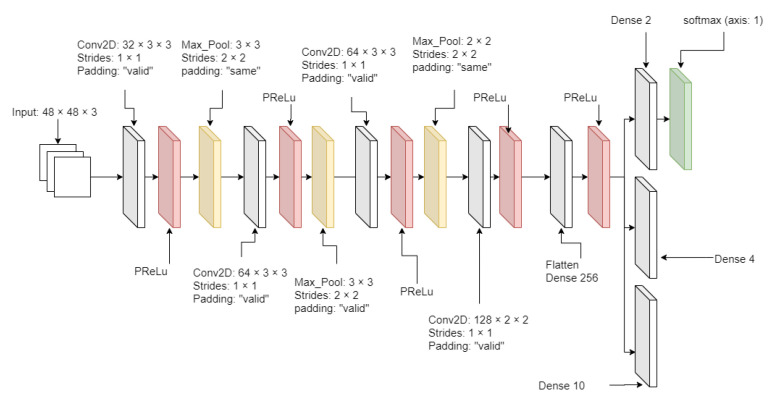
O-Net diagram.

From Figure 2, Figure 3 and Figure 4, MTCNN has P-Net, R-Net, and O-Net. During the face detection, the input image enters P-Net, and R-Net takes P-Net output. O-Net takes the output from R-Net at the end. From P-Net through O-Net, the MTCNN algorithm is getting more complicated and has deeper layers. The number of layers and parameter computation is increased during the process of face detection. All sample images are supposed to enter through these networks to generate the best-detected faces. First, the different-generated-sized input images enter P-Net. P-Net initially chooses the possible face frames from the given input images. R-Net inspects the given initial frames from P-Net, then it removes the face frames specifically which do not meet a threshold score. Finally, O-Net chooses the best face frames from the given output from R-Net. The binary cross-entropy loss from Equation (1) is used to train the MTCNN and is expressed as

(1) $$L_{i}^{det}=-\left[\left[y_{i}^{det}log\left\{p\left(y_{i}^{det}\right)\right\}\right]+\left(1-y_{i}^{det}\right)\left[log\left\{1-p\left(y_{i}^{det}\right)\right\}\right]\right]$$

where $y_{i}^{det}$ is the ground truth of the detected face from input samples, while $p(y_{i}^{det})$ is the probability of predicted and detected faces. Applying a natural logarithm on $p(y_{i}^{det})$ produces an error rate of the predicted face. $y_{i}^{det}log\left\{p\left(y_{i}^{det}\right)\right\}$ is the loss of the detected face, while $\left(1-y_{i}^{det}\right)log\left\{1-\left(py_{i}^{det}\right)\right\}$ is the non-detected face.

In addition to the structure of MTCNN, all activation layers are used by the parametric rectified linear unit (PRelu), which is an improved version of the traditional rectified linear unit (ReLU) or the leaky-ReLU. The PReLU can adjust the slope as a training parameter. The PReLU equation is described as

(2) $$f\left(x_{i}\right)=\left\{\begin{array}{cc} \alpha_{i}x_{i}, & x_{i}\leq 0\\ x_{i}, & x_{i}>0 \end{array}\right.$$

where $x_{i}$ is the input of the *i*-th channel, and $\alpha_{i}$ is a slope value at the negative $x_{i}$ input and a trainable parameter. The slope can be adjusted during the training time. If $\alpha_{i}$ is zero, Equation (2) becomes the traditional ReLU activation function. Or, the Leaky-ReLU activation function if $\alpha_{i}$ is 0.01.

At the end of the MTCNN process as the face extractor, it can generate at least 6800 cropped facial images from a single video clip because each video frame could contain more than 2 or 3 faces. Later, the cropped facial images will be resized after passing through the image resizer.

**Figure 5:**
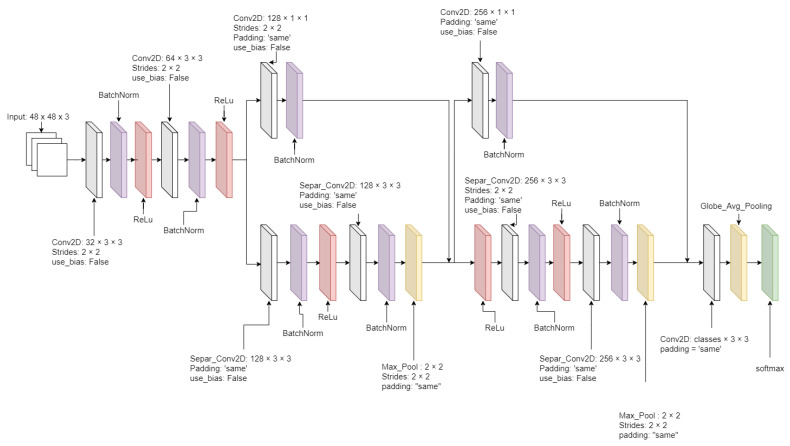
The Xception algorithm diagram.

After the facial images were resized, they met the data segregator as the pre-trained Xception model [23], which segregates into the adequately labeled directories. The pre-trained Xception algorithm was initially trained based on the FER 2013 dataset. The advantage of the Xception algorithm is higher accuracy and faster training speed than algorithms that were tested and showed the results in Section 4.1. The critical element of the Xception algorithm from Figure 5 has a depth-wise separable convolution layer and some shortcut structures. A depth-wise separable convolution layer splits each channel of the input and filter separately, convolves them by each channel, and later split one element of 3 channels to be convoluted until all elements have been convoluted. The depth-wise separable convolution layer reduces the number of parameters compared with the conventional convolution layer. The algorithm also has some shortcut structure that skips over the block of the depth-wise separable convolution layers. Instead, it has the blocks of the conventional convolution layer and the batch normalization layer. The metric loss measurement of the Xception algorithm was applied by the categorical cross-entropy loss function from Equation (3).

The train–test splitter split the group of training and testing datasets in a 70 to 30 ratio. Building the FIT machine made it possible to remove the irrelevant face image, correct the classification of the facial images, and create the intelligence signal process lab (iSPL) dataset, our independent dataset, on a massive scale in a short amount of time. After we created the FIT machine, we built our lab dataset, called the iSPL dataset at Kyungpook National University. In our approach, we collect some additional facial images and merge them into the existing datasets. Finally, we loaded the pre-trained Xception algorithm from the data segregator, trained it by the newly updated dataset, and redeployed it into the pre-trained Xception model. Until the FER system reaches the ideal performance, the FIT machine process will be endlessly repeated. By the end, the pre-trained Xception algorithm becomes the newly improved data segregator and emotion classifier, while the unsupervised learning dataset improved to be the finest supervised learning dataset.

## 4. Experimental Analysis

We evaluated the FER system performance from different existing algorithms such as the simple-CNN, PyFER, MobileNet, Inception V.1, ResNet 50, and the Xception algorithm and datasets such as FER 2013, CK+, iSPL, and the merged datasets. All different algorithms [23,24,25,26,27] were implemented in Keras of Tensorflow with Python v.3.6. The FIT machine was implemented in OpenCV2, os.walk, the pre-trained MTCNN as a face detector, and NumPy.resize() as an image resizer. Every dataset except the FER 2013 dataset split the number of facial images into 70% and 30% respectively for training and testing because the FER 2013 dataset had initially split for training and testing dataset as established earlier. The training conditions for each algorithm were a mini-batch sample size of 32, an initial learning rate of 0.01, the Adam optimizer, the categorical cross-entropy loss function, 150 epochs, 50 variable number of patience, Tensorflow-GPU V. 2.0, Intel(R) Core(TM) i5-10600K CPU@4.10GHz, an 32GB RAM, and GeForce RTX 2070. All training and testing results were applied to the data-augmentation technique and the proposed FIT machine. The FIT machine extracted facial parts and resized all face images into 48 × 48 pixels to fit the size of the FER 2013 dataset. The rest of this section is organized as follows. Section 4.1 shows the experimental results based on the different algorithms and different FER datasets. Section 4.2 describes the experimental results from the individual FER datasets, including the demonstration of how FER 2013, CK+, iSPL datasets were merged.

### 4.1. Choosing the Best of the State-of-Art Algorithms

Different FER datasets are supposed to be tested within the 100 epochs to choose the best algorithm for the FER system. The algorithm performance from Table 1 shows the different numbers of the validation accuracy, loss, and training time. The Xception algorithm showed better performance than most of the algorithms except the residual neural network (ResNet 50) [25] at the FER 2013 dataset training. ResNet 50 shows the competitive performance compared with the validation accuracy of the Xception algorithm. Nonetheless, the Xception algorithm training speed was three times faster than ResNet 50 within 100 epochs. In terms of validation accuracy, loss, and training speed, the Xception algorithm generally performed better than others given the result in Table 1. The validation loss is based on the categorical cross-entropy loss function [28], which is expressed as

(3) $$D\left(L,P\right)=-\sum_{x\in X}L(x)log\{P(x)\}$$

where D(L,P) means a distance between the ground truth label and the probability of labeled class from the predicting model. x∈X means the one emotion classification out of the seven emotion classifications. P(x) in the natural logarithm is the predicted probability of the emotion distribution of class *x*, while L(x) is for the actual binary form of a facial image label.

From Table 1 and Table 2, as the number of total parameters increased until the Xception algorithm, the performance increased. ResNet 50 has the highest total number of parameters from all tested algorithms based on the number of input pixels 48 × 48 from Table 2. ResNet 50 has 25,692,935 total parameters and displayed higher performance than all tested algorithms except the Xception algorithm, which has 208,871 total parameters to train. Despite the small number of total parameters compared to all tested convolution neural networks, the Xception algorithm showed the best performance from the given datasets. The Xception algorithm contains the depth-wise separable convolution layer, which does not increase the additional parameters to train but has a faster training speed than all tested algorithms except the Simpler-CNN algorithm. To test with the magnified and merged datasets containing FER 2013, CK+, and iSPL, the algorithm training speed must be as high as the performing accuracy.

### 4.2. The FER Datasets

**Figure 6:**
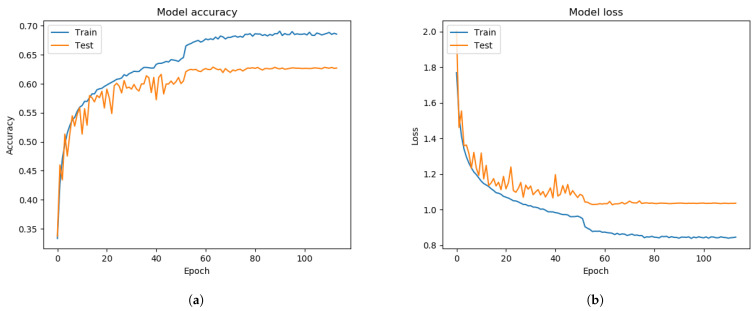
Before applying FIT machine on the FER 2013 dataset. (**a**) model accuracy. (**b**) model loss.

**Figure 7:**
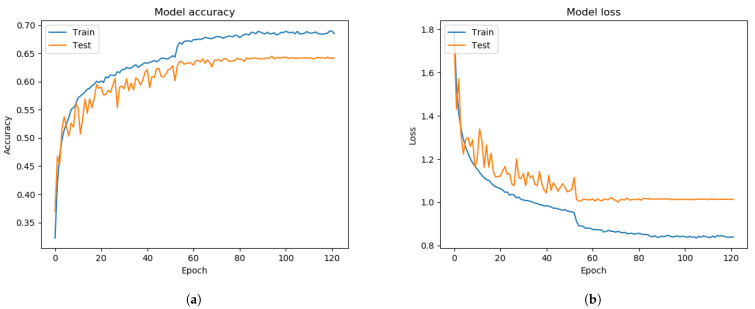
After applying the FIT machine on the FER 2013 dataset. (**a**) model accuracy. (**b**) model loss.

**Figure 8:**
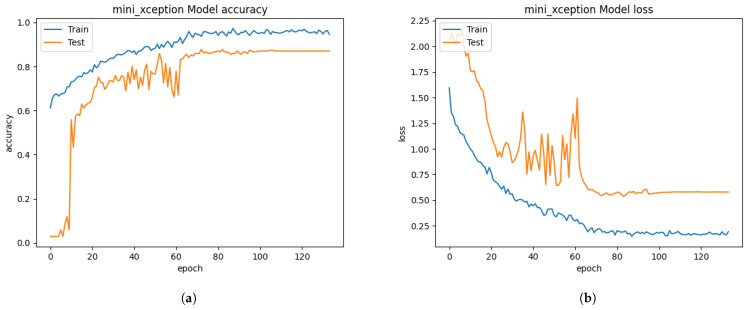
The performance results from the CK+ dataset, which did not extract the facial segment but only resized. (**a**) model accuracy (**b**) model loss.

**Figure 9:**
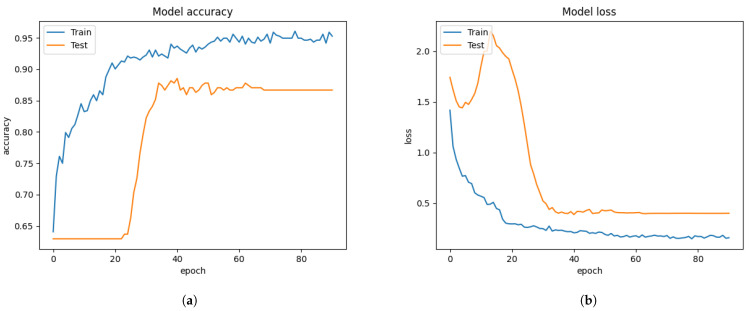
The performance results from the CK+ dataset, which extracted only facial segments from the FIT machine. (**a**) model accuracy (**b**) model loss.

**Figure 10:**
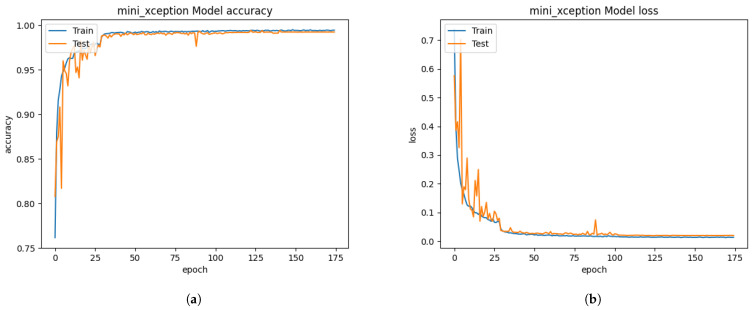
The performance results from the iSPL dataset without applying the FIT machine. (**a**) model accuracy (**b**) model loss.

**Figure 11:**
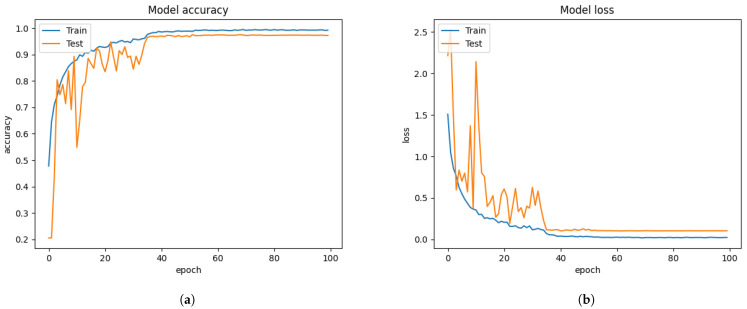
The performance results from the iSPL dataset with the FIT machine. (**a**) model accuracy (**b**) model loss.

**Figure 12:**
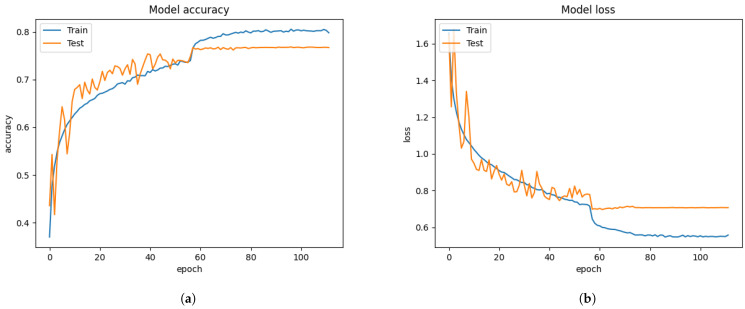
The proposed results from merging FER 2013, CK+, and iSPL datasets operating the FIT machine. (**a**) model accuracy. (**b**) model loss.

In this subsection, all different FER datasets are demonstrated and showed the performance of the Xception algorithm [23]. The epoch variable was 150, and the variable value of the patience was 50. In other words, the training progress of the Xception algorithm stops when no further improvement of validation loss occurs 50 times. The graphs from Figure 6, Figure 7, Figure 8, Figure 9, Figure 10, Figure 11 and Figure 12 demonstrate the Xception algorithm training process within the given individual FER dataset.

**Figure 13:**
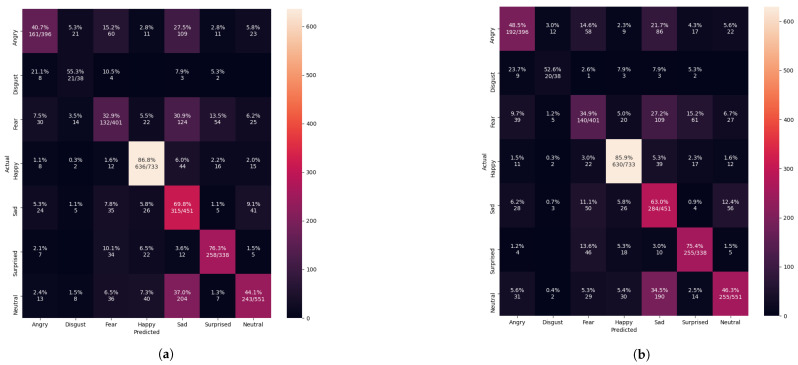
Evaluating the pre-trained Xcepetion algorithm after training with only the FER 2013 dataset. (**a**) Confusion matrix evaluation before applying with the FIT machine. (**b**) Confusion matrix evaluation after applying with the FIT machine.

**Figure 14:**
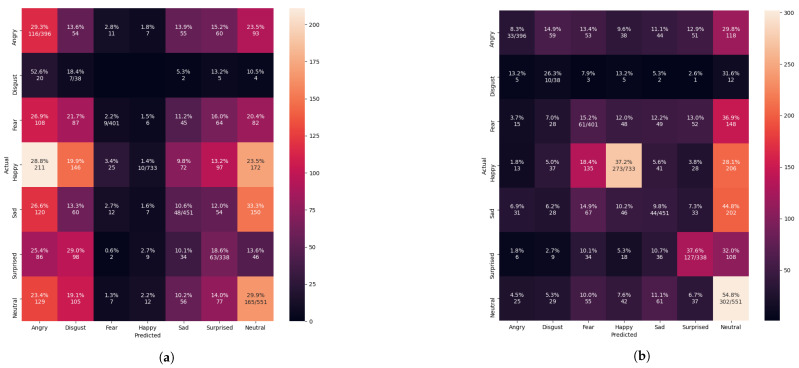
Evaluating the pre-trained Xception algorithm performance after training with the CK+ dataset. (**a**) Confusion matrix evaluation before applying with the FIT machine. (**b**) Confusion matrix evaluation after applying with the FIT machine.

**Figure 15:**
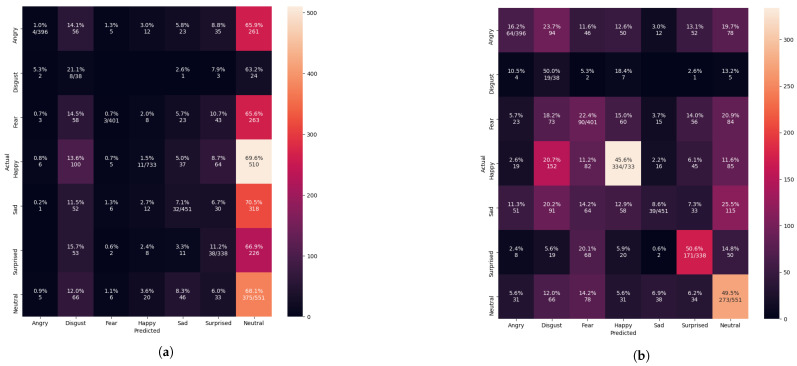
Evaluating the pre-trained Xcepetion algorithm after training with the iSPL dataset. (**a**) Confusion matrix evaluation before the FIT machine result (**b**) Confusion matrix evaluation after the FIT machine result.

**Figure 16:**
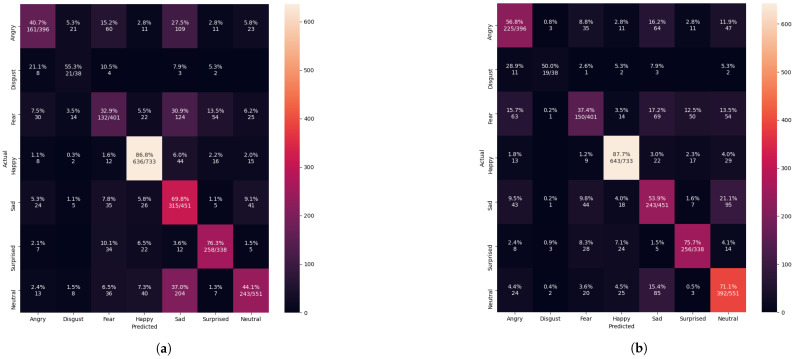
The Xception algorithm was evaluated performing a confusion matrix after training the FER 2013 dataset or the merged datasets of the FER 2013, the CK+ and the iSPL datasets (**a**) the confusion matrix evaluation after training with only the FER 2013 dataset but without applying the FIT machine. (**b**) confusion matrix evaluation after training with the merged dataset and the FIT machine.

The confusion matrix evaluation results from Figure 13, Figure 14, Figure 15 and Figure 16 show the performance of the pre-trained Xception algorithm, which had been initially trained with the given individual FER dataset. The unseen private testing dataset was tested with the pre-trained Xception algorithm. The unseen private testing dataset was a part of the FER 2013 dataset but not used for the Xception model training. We also applied the FIT machine on the unseen private testing dataset to remove some irrelevant facial images which were unfit for the model testing. These figures showed how the pre-trained Xception algorithm based on the individual FER dataset detects new emotional faces properly. The results from Table 3, Table 4, Table 5, and Table 6 display the summary of Figure 13, Figure 14, Figure 15 and Figure 16. The pre-trained Xception algorithm performs well on the unseen private testing dataset if the test samples were densely populated from the left top corner to the confusion matrix figures’ right bottom corner. The results from these figures represented real-time camera testing. The result from Table 3, Table 4, Table 5, and Table 6 was computed based on the following Equations (4)–(6) as parts of the categorical evaluation metrics:

(4) $$P=\frac{TP}{TP+FP}$$

(5) $$R=\frac{TP}{TP+FN}$$

(6) $$F_{1}=2\times \frac{P\times R}{P+R}$$

where TP, FP, FN, *P*, *R*, and $F_{1}$ respectively stand for true positive, false positive, false negative, precision, recall, and f-1 score. Precision from Equation (4) is the percentage of accuracy from the true positive detection TP out of the total predicted positive TP+FP. Recall that Equation (5) is based on the true positive detection TP out of the total actual positive TP+FN. Wang et al. [29] summarized those equations, and the f1-score from Equation (6) is for balancing the precision and recall when it comes to uneven dataset distribution.

#### 4.2.1. The FER 2013 Dataset

The FER 2013 dataset was obtained in a comma-separable values (CSV) format, where was very hard to perceive the facial emotion from a series of pixel values. FER 2013 has 28,655 facial images for training and around 7166 facial image files for the testing. The public testing dataset has 3582 facial images, while the private testing dataset has 3584. The total number of facial images in the FER 2013 dataset is around 35,821. Each facial image size is 48 × 48 pixels, and there are seven different labeled classified emotions such as anger, disgust, fear, happiness, neutral, sad, and surprised. As mentioned in the dataset reliability in Section 2, we converted the CSV file into the images. All faces in the picture have various poses of front, side, half-side, or half-rotated. By inspecting the dataset meticulously, 83 irrelevant facial images were found. Others were not relevant to the adequately labeled directory. These problems could be resolved by operating the FIT machine, removing the irrelevant facial images, and reorganized the misplaced face images.

From Figure 6, we trained and tested the Xception algorithm performance without the FIT machine using the FER 2013 dataset. The performance of the FER system showed 60.99% of validation accuracy and 1.1529 of the validating categorical cross-entropy error distance as Equation (3) during the training of the Xception algorithm from Figure 6. Converting the CSV file into images allowed for quick inspection of some irrelevant or mis-labeled face images. This malicious face data is supposed to be removed or correctly labeled before training the Xception algorithm. Otherwise, the pre-trained model of the Xception algorithm showed poor performance.

In our approaches, clearing out the irrelevant images and the facial images correctly labeled by using the FIT machine, the FER system performance slightly improved by 3% validation accuracy as compared with the results from Figure 6 and Figure 7. We also discovered that the gap between training and testing graphs was reduced. The Xception algorithm with the sole FER 2013 reached 63.99% of validation accuracy during the training process from Figure 7. The face images of the FER 2013 dataset were mostly and appropriately prepossessed, while a small number of them were not, so the performance was slightly improved.

Figure 13 shows the pre-trained Xception algorithm using unseen private testing dataset from FER 2013. The confusion matrix results from Figure 13 confirmed to have 61.7% precision from Equation (4), 58.8% recall from Equation (5), and 59.4% f1-score from Equation (6) before we applied the FIT machine. After applying the FIT machine, Table 3 showed the result of 63% precision, 61% the recall, and 61% f1-score. However, removing some irrelevant facial images could potentially reduce the number of the available facial images, and relabeling the facial images could cause the imbalanced number of the facial images in each label-categorized directory. From Figure 13 and Table 3, although we improved the overall classification performance, some performance was reduced. To substitute the missing facial images, any small dataset, especially CK+, had to be merged into the FER 2013 dataset.

#### 4.2.2. The CK+ Dataset

The CK+ dataset consists of 920 trainable images and eight classified emotions: anger, disgust, contempt, fear, happiness, neutral, sadness, and surprise. All image sizes are 640 × 490 pixels. The face images of the CK+ dataset have only frontal face pose and a small number of face pictures compared to the FER 2013 dataset. All volunteer face images have larger background segmentation than their facial segmentation. Without resizing to the 48 × 48 pixels, the pre-trained Xception algorithm based on the 640 × 490 pixels image was unable to test due to the mismatching size of the unseen private testing dataset, which is 48 × 48 pixels. Moreover, the Xception algorithm is not flexible with a different size of the input images. Initially, we only resized those images into 48 × 48 pixels without properly extracting the facial segments. From Figure 8, the training process of the Xception algorithm under the CK+ dataset showed much better results than the training of Xception algorithm from FER 2013 dataset. The graph result from Figure 8 had 86.66% of validation accuracy and 0.5382 of the validating categorical cross-entropy error distance.

Nevertheless, the unseen private testing dataset showed worse performance than the training with the FER 2013 dataset from Figure 14a and Table 4. The results of the precision, recall, and f1-score were 17%, 14%, and 12.7%, respectively. Each face image was supposed to be adequately cropped and resized to improve the system performance. To improve the system performance from resizing the images, operating the FIT machine is necessary to remove the large background segmentation.

The FIT machine matched the sizes of the unseen private dataset and removed the background segmentation. After extracting and resizing all facial segments, the Xception algorithm for the FER system was adequately trained and tested. The graph result from Figure 9 showed the 88.52% of validation accuracy and 0.3862 of validation loss, and the result of the confusion matrix evaluation from the unseen private testing dataset, which significantly improved to 32% precision, 27% recall, and 22% f1-score as shown in Figure 14b and Table 4. Although the newly trained Xception algorithm was improved the system performance under the influence of the CK+ dataset and the FIT machine, it still led to worse performance than FER 2013 dataset. In our camera test, the pre-trained Xception algorithm from CK+ dataset merely detects facial emotions but performed well on the FER 2013, so these results from Figure 14 similarly represent our camera test. The small number of facial images in the CK+ dataset to train even with data augmentation still led to a biased result during the model training process.

Moreover, we previously split 90 to 10 training and testing with 10-fold cross-validation [30], which could lead to better performance than splitting 70 to 30 for the ratio of training and testing datasets. Regardless of these methods, the pre-trained Xception algorithm has some problems for new face detection. The 10-fold cross-validation experiment displayed bias evaluation among real-world evaluation. Our goal of the FER research is to apply for real-world testing to resolve the insufficient number of face emotion data to train and test. Therefore, training the Xception algorithm with the CK+ dataset caused defective performance from the unseen private testing dataset and failed to generalize the facial emotion in the real world due to its lack of facial emotion variations.

#### 4.2.3. The iSPL Dataset

Before creating the FIT machine, all video clips had different playtime lengths and different sizes of resolutions. Some had 640 × 450 pixels of the video clip resolution, but others had 1920 × 1080 pixels. Some video clips scenes do not have any faces or contain more than two faces in different positions. Most CNNs, including the Xception algorithm, do not have flexibility in the input image different sizes and cause a technical program error due to mismatching the input size. Training the Xception algorithm with the non-identical size input image would be impossible without the FIT machine. Although we managed to resize all video frames, the resized images without proper face extraction for training and testing could degrade the FER dataset quality. The 2-min video clips could have more than 7200 frames if a video clip has 60 frames per second. Manually collecting the facial images from the video clips would waste our time and energy.

Initially, we resized those images into 48 × 48 pixels to reduce the training computation without applying the FIT machine. Abnormally, the performance of the Xception algorithm from Figure 10 reached 99.22% of validation accuracy and 0.0188 of validation loss. We have discovered that even a bad dataset could show outstanding performance to the Xception algorithm. Testing with the unseen private testing dataset from Figure 15a and Table 5 showed how biased results affect the FER system performance. The result of the precision, recall and f1-score from Table 5 was 16.32%, 16.20%, 9.92% respectively. Training the Xception algorithm from the iSPL dataset without applying the FIT machine might perform well on training and validating, but it hardly detected the strong emotions from new faces in real-time testing.

With the FIT machine, iSPL dataset [21] was correctly created. iSPL dataset is used to compare the state-of-art algorithm performance or add more available facial images into original datasets. All people in the dataset emotionally expressed during the real-time critical events. The dataset contained 8173 facial images, and each person’s facial emotion was sequentially recorded. The size of each iSPL dataset facial image was 48 × 48 pixels, and it contains seven categorical emotions: anger, disgust, fear, happiness, neutral, sadness, and surprise. All facial images were converted into gray-scale to reduce the input channel. If one classified label had insufficient facial images as less than 1000 facial images, the trained FER system would not identify that specific emotion correctly. Therefore, we stored 1000 facial images into each directory as we kept balancing the number of facial images in every labeled directory.

Training the Xception algorithm with the iSPL dataset and applying it to the FIT machine reached 97% validation accuracy from Figure 11 but had 3% less validation accuracy than Figure 10’s result. On the other hand, the confusion matrix evaluation from Figure 15b and Table 5 had 17% improvement compared to the iSPL dataset without the FIT machine. From Table 4 and Table 5, the confusion matrix evaluation under condition of the iSPL dataset with the FIT machine had 6% additional improvement from the CK+ dataset. Still, the pre-trained Xception algorithm with the iSPL dataset did not outperform the FER 2013 dataset from Table 3. The small-sized FER dataset such as CK+ or iSPL dataset performed poorly in the real-time testing according to results from Table 4 and Table 5. Rather than using the singular CK+ or iSPL dataset, we were deterministic that if CK+ and iSPL datasets could be merged into the FER 2013 dataset and substituted to our missing facial images of FER 2013 dataset if we could improve the performance further by increasing the number of face images to train and test.

#### 4.2.4. The Merged Dataset

Ultimately, merging the different datasets [31] used to be a tedious processes before the creation of the FIT machine because each dataset has its unique transformation of facial images. For example, CK+ has a larger background segment than the facial segment. Unlike CK+, FER 2013 has a smaller portion of a background segment than the face segment but contained irrelevant face images. Merging them without standardizing those two different datasets would render poor performance during the model training or have a software problem due to the mismatching input size. Also, manually cropping and resizing the massive number of facial images seemed quite exhausting before training the Xception algorithm.

**Figure 17:**
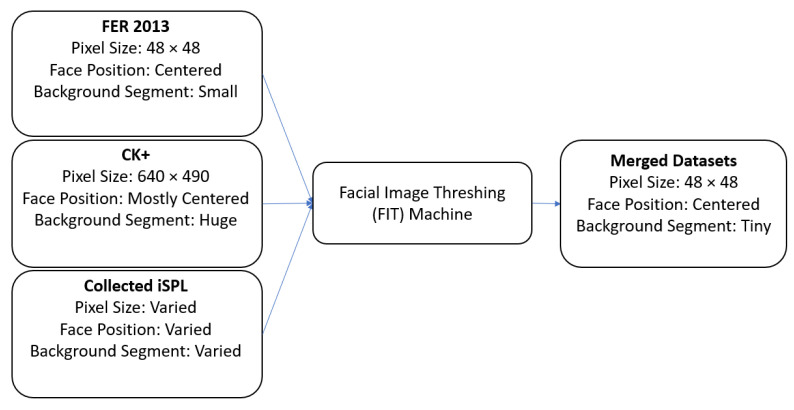
Merging the CK+, the FER 2013, and the iSPL datasets by using the FIT machine.

**Figure 18:**
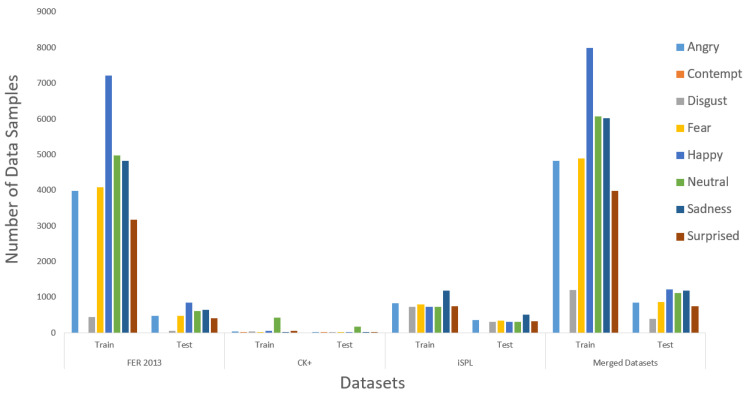
The Data Distribution.

To merge three different datasets, we applied the FIT machines to all existing datasets from Figure 17. After all, facial images from CK+ and iSPL dataset were cropped correctly and resized as they fit into the FER 2013 dataset, the split train and test program randomly split into training and testing datasets. The distributing ratio for the training and testing dataset was 70 to 30 according to Figure 18. The contempt-labeled category has been omitted due to a few facial images that caused the merged dataset’s distribution to become imbalanced and hardly detected the contempt emotion. Finally, the three different datasets were merged before we started the training process of the Xception algorithm.

After the Xception algorithm was trained with the merged datasets, the graph results from Figure 12 showed better performance than training with only the FER 2013 dataset from Figure 6. From Figure 12, the validation accuracy of the merged datasets and Xception algorithm reached 77.94%, while the conventional approach reached 60.99% from Figure 6.

From Table 6, the merged datasets showed improvement in facial classification, and the system precision, recall, and f1-score were reached 66.62%, 66.88%, and 66.67%. The testing samples from Figure 16 were more densely populated at the left-leaning diagonal line compared to Figure 14 and Figure 15. The FIT machine made it possible to create the merged dataset without concerning the unique FER dataset transformation. We improved the validation accuracy by 16.95% over the FER 2013 dataset from Figure 6. The estimated 5% improvement from Table 6 means that the pre-trained algorithm correctly captures more face emotions from the camera test and segregates more face emotions within correct labeled classifications compared to the conventional approach from Figure 16a. However, the samples from disgust, sadness, and surprise classification results were slightly dropped due to the poor training of the Xception algorithm. Further improvement is required by operating the FIT machine with the newly trained Xception algorithm.

From Table 7, the optimizer, learning rate, momentum, and epsilon were used as the hyperparameters. We analyzed the FER system performance based on different optimizers such as Adam and the stochastic gradient descent (SGD) with different learning rates. The learning rates used for our situation are 1, 0.1, 0.01, 0.001, and 0.0001. During the Xception algorithm training, the learning rate was automatically reduced and reaches the optimal gradient point. The momentum as the hyper-parameter of the SGD optimizer, we used 0.3, 0.6, and 0.9 values. In the case of epsilon as the hyper-parameter of Adam optimizer, we used 1.0 × 10^−6^, 1.0 × 10^−7^, 1.0 × 10^−8^, and 1.0 × 10^−9^ values. The SGD with a learning rate of 1 showed the worst performance due to the high learning rate used in the Xception algorithm. The epsilon as 1.0 × 10^−6^ showed better performance in validation loss and the system provides accurate results if the dataset arrangement were perfectly segregated.

In contrast, some researchers argue that data augmentation resolves the lack of facial samples for training. Even with the data-augmentation technique, training with a small dataset hardly detects new face emotions. We always use data augmentation to train all algorithms regardless of the training sample size as we prevent the possible overfitting problem during the deep neural network training. However, we still failed to generalize the outside world facial emotions. The data augmentation does not augment the number of facial images nor a complete solution of generalizing the representation for the outside world facial emotions. Through these experiments, operating the FIT machine could become another option than the data-augmentation technique.

## 5. Conclusions

This paper proposed a FIT machine for the FER dataset manager as it could remove any irrelevant data, reorganize existing datasets, collect an additional dataset, and merge all existing datasets. The FIT machine makes an unsupervised learning dataset to be a supervised learning dataset. Creating facial images for the FER dataset or a facial-related dataset could be cheaper for many FER developers with the FIT machine. The FIT machine could be useful for many researchers and developers who want to create an independent or customized FER dataset to enhance the FER system performance even faster.

## Figures and Tables

**Table 1:** The performance of different algorithms and datasets as all given datasets were applied by data augmentation and FIT machine.

Algorithm	Performance								
	FER 2013			CK+			iSPL		
	Accuracy (%)	Loss	Training Time (s)	Accuracy (%)	Loss	Training Time (s)	Accuracy (%)	Loss	Training Time (s)
Simple-CNN	59.18	1.0689	1932	85.24	0.4425	64	95.02	0.1622	443
PyFER	60.16	1.0942	1859	82.29	0.7632	75	95.15	0.2032	600
MobileNet	61.13	1.0351	2867	80.81	0.6469	119	97.31	0.0966	775
Inception V. 1	61.12	1.0563	4504	87.45	0.492	201	96.57	0.1628	1037
ResNet 50	64.22	1.0065	9938	85.61	0.519	323	97.72	0.0976	2219
Xception	63.99	1.0393	3234	88.52	0.3862	96	97.92	0.0778	653

**Table 2:** The number of parameters from different algorithms.

Algorithms	Total Parameters	Trainable Parameters	Non-Trainable Parameters
Simple-CNN	642,935	651,463	1472
PyFER	3,934,199	3,933,239	960
MobileNet	3,246,407	3,224,519	21,888
Inception v.1	6,386,373	6,386,373	0
ResNet 50	25,692,935	25,639,815	53,120
Xception	208,871	206,375	2496

**Table 3:** The summarized performance of Figure 13.

Datasets	Precision (%)	Recall (%)	F1_Score (%)
Only the FER 2013 before the FIT Machine	61.6532	58.7689	59.4004
Only the FER 2013 after the FIT Machine	63.0118	61.0729	61.0932

**Table 4:** The summarized results of Figure 14.

Datasets	Precision (%)	Recall (%)	F1_Score (%)
Only the CK+ before the FIT Machine	17.3592	14.3741	12.7031
Only the CK+ after the FIT Machine	32.0083	27.8542	22.4103

**Table 5:** The summarized results of Figure 15.

Datasets	Precision (%)	Recall (%)	F1_Score (%)
Only the iSPL+ before the FIT Machine	16.3159	16.1966	9.9174
Only the iSPL+ after the FIT Machine	39.4900	33.2531	33.9923

**Table 6:** The summarized results of Figure 16.

Datasets	Precision (%)	Recall (%)	F1-Score (%)
Only the FER 2013 after the FIT Machine	63.0118	61.0729	61.0932
The Merged Datasets	66.6236	66.8845	66.6779

**Table 7:** The results of conducting the different hyperparameters experiments from the merged datasets.

**Optimizer**	**Initial-LR**	**Momentum**	**V. Accuracy (%)**	**V. Loss**	**Precision (%)**	**Recall (%)**	**F1-Score (%)**
SGD	1	0.0	0.1298	Infinity	0.0000	0.0000	0.0000
SGD	0.1	0.0	76.5550	0.6775	64.5930	65.1306	64.6584
SGD	0.01	0.0	74.9003	0.7127	65.0916	65.4057	65.0358
SGD	0.001	0.0	70.2153	0.8621	58.8519	60.4195	59.2480
SGD	0.0001	0.0	64.8724	0.9844	55.4958	57.1182	55.9766
SGD	0.01	0.3	75.1196	0.7274	64.1630	64.6492	64.1351
SGD	0.01	0.6	75.0598	0.7250	63.8704	63.9528	55.9766
SGD	0.01	0.9	76.1762	0.6911	55.4958	57.1182	55.9766
**Optimizer**	**Initial-LR**	**Epsilon**	**V. Accuracy (%)**	**V. Loss**	**Precision (%)**	**Recall (%)**	**F1-Score (%)**
Adam	1	1.0 × 10^−7^	20.2751	2.3080	6.3535	25.2063	10.1489
Adam	0.1	1.0 × 10^−7^	58.1738	1.1357	51.2177	53.3356	51.9438
Adam	0.01	1.0 × 10^−7^	76.2360	0.6665	64.6893	65.0275	64.6610
Adam	0.001	1.0 × 10^−7^	77.0933	0.6656	66.4377	66.7125	66.3362
Adam	0.0001	1.0 × 10^−7^	73.8237	0.7615	61.8960	62.5859	62.1137
Adam	0.001	1.0 × 10^−6^	76.6148	0.6603	65.3901	65.6808	65.4316
Adam	0.001	1.0 × 10^−8^	77.2727	0.6732	65.8512	66.2654	65.9031
Adam	0.001	1.0 × 10^−9^	77.1730	0.6605	66.3113	66.5061	66.3348

## Data Availability

Not applicable.
